# Porous Microparticles of Corn Starch as Bio-Carriers for Chia Oil

**DOI:** 10.3390/foods11244022

**Published:** 2022-12-13

**Authors:** Roxana V. Piloni, M. Gabriela Bordón, Gabriela N. Barrera, Marcela L. Martínez, Pablo D. Ribotta

**Affiliations:** 1 Instituto de Ciencia y Tecnología de los Alimentos Córdoba (ICYTAC-CONICET), Juan Filloy S/N, Córdoba X5000HUA, Argentina; 2 Instituto de Ciencia y Tecnología de los Alimentos (ICTA-FCEFyN), Universidad Nacional de Córdoba (UNC), Av. Vélez Sarsfield 1611, Córdoba X5016GCA, Argentina; 3 Departamento de Química Industrial y Aplicada, Facultad de Ciencias Exactas, Físicas y Naturales (FCEFyN), Universidad Nacional de Córdoba (UNC), Av. Vélez Sarsfield 1611, Córdoba X5016GCA, Argentina; 4 Instituto Multidisciplinario de Biología Vegetal (IMBIV-CONICET), Av. Vélez Sarsfield 1611, Córdoba X5016GCA, Argentina

**Keywords:** porous starch, impregnation, chia oil, omega-3, functional food

## Abstract

Native corn starch and pretreated corn starch were treated with α-amylase, glucoamylase and mixtures of both to generate starches with high porosity with conserved granular structure. Porous starches were characterized; particle size distribution analysis, nitrogen adsorption-desorption analysis, scanning electron microscopy, water and oil adsorption capacity, differential scanning calorimeter, X-ray diffraction and damaged starch techniques were used. The α-amylase/glucoamylase mixture at the highest dose was the best treatment to generate porous starches with interesting adsorption capacity and granular structure conservation. Selected starches were impregnated with chia oil using a vacuum. Pretreated corn starch modified with the α-amylase/glucoamylase mixture showed no significant differences on impregnation capacity compared with native starch with a similar enzyme treatment. The highest oxidative stability was achieved with pretreated porous starch impregnated with 10 to 25% chia oil, compared with the bulk oil (5.37 to 4.72 and 2.58 h, respectively). Results have demonstrated that vacuum impregnation could be a potential technique for the incorporation of oil in porous structures based on starch and porous starches obtained by enzymatic hydrolysis are a promising material for the incorporation and protection of oils susceptible to oxidation.

## 1. Introduction

Functional food has shown benefits for human health beyond basic nutrition, either by eliminating harmful components or by enriching food with bio-active components [1,2]. Multiple bio-active molecules have been used to enrich food products, such as vitamins, peptides, amino acids, antioxidants, polyunsaturated fatty acids and dietary fiber.

A diet rich in polyunsaturated fatty acids (PUFAs) has been linked to the prevention of certain noncommunicable illnesses, such as coronary artery disease, diabetes and cancer, playing an essential role in physiology during fetal and infant growth [3,4]. PUFAs can be obtained not only from fish but also from vegetable sources, for instance linseed, chia, sunflower and olive oil. Chia seed (*Salvia hispanica* L.) contains about 0.25 and 0.38 g oil/g seed in which α-linolenic (omega-3) and α-linoleic (omega-6) acids are present in high amounts (61–70 and 18–22%, respectively). It also has a raised level of proteins, fibers and antioxidants such as tocopherols and polyphenols (mainly chlorogenic and caffeic acids) [5]. In this sense, enriching staple foods, such as bread, dry pasta, milk, chocolate and hamburger, with omega-3 fatty acids provides an alternative to increase its consumption, especially by people not used to eating seeds or fish.

Despite the health benefits associated with a regular diet containing omega-3-rich oils, their polyunsaturated structure makes this oil highly susceptible to degradation reactions when exposed to air, humidity, light or temperature [6]. Different strategies have been applied to stabilize chia oil, the most commonly used being the addition of natural or synthetic antioxidants and microencapsulation in different biopolymer matrices [7,8,9,10]. Alternatively, a bio-based carrier method may offer some advantages by incorporating and protecting susceptible oils. Carriers based on starch, whey protein, egg protein and alginate have been used and studied for oil encapsulation [11,12,13,14]. Starch, especially corn starch, is a material widely used industrially in several areas due to its high production worldwide, low price, relative ease of isolation in its pure form from plant source, innocuousness and biocompatibility [15]. Through physical, chemical or enzymatic treatments, it is possible to develop porous starch structures with high adsorption capacity for its application as a shell material to improve the stability and water solubility of compounds.

Enzymatic treatments are more suitable because they generate fairly regular pores. As relatively mild treatments, they keep intact the granule structure [16]. Many enzymes have been studied for this purpose, including α- and β-amylases, glucoamylase, cyclodextrin-glycosyltransferase and isoamylase. Zhang et al. [17], Han et al. [18] and Dura et al. [19] studied the effect of α-amylase and glucoamylase on corn starch, showing very uniform porosity. Guo et al. [20] studied the effect of other enzymes such as glycosyltransferase and branching enzyme over starch from different botanical sources, achieving a uniform high-porosity starch matrix.

A challenging aspect in the use of porous starch is the incorporation of a bio-active compound into the structure. So far, research has been carried out to describe the production of porous microparticles loaded with polyunsaturated oils by means of passive absorption [21] and supercritical fluids [22]. Therefore, new, better and cheaper methodologies to facilitate the incorporation of these components into microparticles are required. Vacuum impregnation (VI) is a potential methodology due to its advantages over the other techniques; it is safer since no high-pressure systems are needed, impregnation times are shorter compared with those of passive methods and it is cheaper compared with supercritical fluid approaches. However, as far as we know, no research has been conducted on the use of VI to incorporate a bio-active compound into porous microstructures.

The objective of this research was to analyze the potential of porous corn starch to incorporate and protect chia oil. Accordingly, different enzymatic treatments were evaluated using native and pretreated corn starch at sub-gelatinization temperature to obtain high-porosity granular structures. Chia oil was then added to porous starch by vacuum impregnation and oil stability was evaluated.

## 2. Materials and Methods

### 2.1. Reagents

Food-grade corn starch (NS) was used (Distribuidora Nicco, Córdoba, Argentina). Pretreated starch (PS) at sub-gelatinization temperature was produced by placing equal amounts of corn starch and distilled water, heating for 10 min at 50 °C in a water bath and drying for 48 h at 40 °C. Disodium hydrogen phosphate (Cicarelli, Santa Fe, Argentina), sodium dihydrogen phosphate (Cicarelli, Santa Fe, Argentina), citric acid (Anedra, Buenos Aires, Argentina), sodium acetate (Anedra, Buenos Aires, Argentina), acetic acid (Sintorgan, Buenos Aires, Argentina) and sodium hydroxide (Cicarelli, Santa Fe, Argentina) were analytical reagents. Food-grade α-amylase (5000 SKB/g, here referred to as AM) and glucoamylase (3300 U/g, referred to as GAM) were purchased from Granotec (Buenos Aires, Argentina). All buffers were prepared with deionized water. 

Chia oil was obtained from seeds from the province of Salta, Argentina (Nutracéutica Sturla SRL, Buenos Aires, Argentina), using optimized conditions described by Martinez et al. [23]. Acidity index (IA), peroxide index (PI), specific extinction coefficients K232 and K270 and oxidative stability as induction time (IT, h) were determined for chia oil characterization, according to the standard methods of AOCS [24]. The fatty acid profile was analyzed by gas chromatography (GC) using the methodology proposed by Martinez et al. [25].

### 2.2. Porous Starch Preparation

Porous starch was prepared following Zhang et al. experimental method [17], with some modifications. Five grams of NS or PS were weighed in 50 mL centrifuge tubes. One AM dose and two GAM doses, a lower (GAM-L) and a higher one (GAM-H), were considered according to previous experiments: AM = 100 U/g, GAM-L = 66 U/g and GAM-H = 200 U/g. In addition, mixtures of both were tested (AM/GAM-L = 100:10 U/g and AM/GAM-H = 100:30 U/g). Enzymes were added to the tubes along with 25 mL of buffer solution (phosphate buffer pH 6 for AM, acetate buffer pH 4 for GAM and phosphate citric acid buffer pH 5.5 for AM/GAM experiments) and they were placed in a thermostatic orbital stirring bath (SHZ-88, Arcano, Buenos Aires, Argentina). The reaction was carried out at 50 °C, shaking at 80 rpm for 12 h. Then, sodium hydroxide solution (0.1 M) was added to adjust the pH value to 10 to inactivate the enzymes. Finally, samples were centrifuged at 7000 rpm (Sorvall ST 40R, ThermoScientific, Waltham, MA, USA), the supernatant was discarded and the starch was washed twice with distilled water. The product was dried for 48 h at 45 °C and milled for further use. Several batches were produced for each sample.

### 2.3. Particle Size Distribution

The size distribution of starch particles was determined by laser diffraction with an LA 950V2 Horiba (Kyoto, Japan) analyzer at 25 °C based on Bordón et al. [26]. The refractive index used was 1.34. The D[3,2] (Sauter) and D[4,3] (De Broucker) mean diameters (Equations (1) and (2)) were determined. The median of the volume-weighted size distribution of starch particles, D_V50_, D_V90_ and D_V10_, was also found.

D[4,3] = ∑ni.Di^3^/ = ∑ni.Di^2^, (1)

D[3,2] = ∑ni.Di^4^/ = ∑ni.Di^3^, (2)

### 2.4. Scanning Electron Microscopy (SEM) Characterization

Sample morphology was determined by field emission scanning electron microscopy (FE-SEM Σigma, Carl Zeiss, Oberkochen, Germany) with an in lens detector at 10 kV. Previously, the samples were metalized with a 20 nm layer of Cr before measuring.

### 2.5. Specific Surface Area Analysis

Pore size distribution and specific surface area were determined using a high-performance adsorption analyzer ASAP2020 Plus (Micromeritics Instrument Corporation, Norcross, GA, USA) with nitrogen adsorption at 77 K. The adsorption–desorption isotherms were established with high-purity nitrogen (>99.99%). One gram of each starch sample was dehydrated at 80 °C for 16 h and degassed at 100 °C for 10 h before measuring. Relative pressures ranged (P/P0) from 0.02 to 0.99. Specific surface area was calculated using the Brunauer–Emmett–Teller (BET) method, and pore size distribution was analyzed with Barrett–Joyner–Halenda (BJH) method.

### 2.6. Water and Oil Adsorption Capacity of Porous Starch Samples

The water and oil absorptive capacity of native and porous starch was determined following the procedure reported by Yousif et al. [27], with modifications. Starch samples (0.1 g) were suspended into 1 mL of solvent (water or sunflower oil) in a 50 mL centrifuge tube at room temperature. The tube was shaken with a vortex for 30 min, centrifuged at 10,000 rpm for 10 min and the supernatant was discarded. The precipitate starch was weighed and the absorption capacity was calculated as mass (g) of impregnated starch by mass (g) of dry starch. Analyses were performed in duplicate.

### 2.7. Starch Thermal Analysis

The thermal analysis of the starch was carried out using a differential scanning calorimeter (DSC 823e, Mettler Toledo, Greifensee, Switzerland); thermograms were evaluated by STARe software (V 9.00, Mettler Toledo, Greifensee, Switzerland). Starch samples (15 mg) were weighed and placed into 100 mL aluminum pans with 30 µL of deionized water. These pans were then hermetically sealed and allowed to stand for 24 h at room temperature before DSC analysis. Pans were subjected to the following DSC conditions: 5 min at 25 °C and heating until 120 °C at 10 °C/min. An empty and sealed pan was used as a reference for all measurements. Thermal behaviors were characterized through onset temperature (T_o_), conclusion temperature (T_c_) and gelatinization enthalpy (ΔH). Analyses were performed in triplicate.

### 2.8. X-ray Diffraction (XRD) and Damaged Starch Determination of Selected Porous Starch

XRD patterns of selected starches were carried out by a diffractometer Philips PW1800 (USA). The patterns were obtained with Cu kα radiation (λ = 0.154 nm) and the operated conditions were: 30 mA, 40 kV, 2–40° scanning region (2θ) and 0.01 °/s scanning speed. Spectrum fitting and crystallinity percentage were calculated using PeakFit (version 4.12, Seasolve software, Inc., San Jose, CA, USA) [28]. The damaged starch of selected samples was estimated enzymatically, in duplicate, following the American Association of Cereal Chemistry (AACC) methods 76-30A and 80-60 [29,30].

### 2.9. Oil Incorporation and Characterization of Impregnated Starch

#### 2.9.1. Chia Oil Impregnation

The impregnation conditions were based on previous studies with different matrixes and bio-active compounds [31]. In the present work, the conditions were adjusted by preliminaries assays due to the bio-active compound hydrophobicity (data not shown).

Incorporation of chia oil in selected samples was carried out via the vacuum impregnation (VI) technique, using an ultravacuum pump Dosivac DVR280 (Buenos Aires, Argentina) with a rated vacuum of 0.020 bar. Two grams of porous starch was put in contact with increasing percentages of chia oil (10–55% *w/w*) and kept at 30 °C (tubes immersed in a water bath) and shaken during 30 min to achieve a homogeneous mixture. Then, the porous starch/oil mixtures were placed in a sealed flask and vacuum was applied for 15 min (room temperature, ~24 °C). Pressure was reestablished and samples were saved in sealed bags at −40 °C for further characterization. Analyses were performed in duplicate.

#### 2.9.2. Colorimetric Determination

Impregnated starch color was determined with a surface spectrophotometer (CM600d Konica-Minolta, Tokio, Japan). Samples were placed homogeneously over a Petri plate and covered with a low reflectance glass. Three readings were taken for each sample and color was assessed according to the CIELAB scale, L* (lightness), a*(redness-greenness) and b*(yellowness-blueness). Measurements were performed in triplicate.

Whiteness index (WI) and yellowness index (YI) were calculated using Equations (3) and (4), respectively [32]. Linear regressions of these indexes were performed to assess the effectiveness of VI methodology.

WI = L − 3b,(3)

YI = 142.86 b/L,(4)

#### 2.9.3. Oil Oxidative Stability Analysis under Accelerated Conditions

Oxidative stability was determined in duplicate using the Rancimat accelerated oxidation method (Metrohm, Herisau, Switzerland) according to González et al. [7] with slight modifications. Impregnated starches (0.5–1.0 g) and chia oil (as a control) were exposed to 100 °C with a fixed air flow of 20 mL/min. The volatile oxidation products were absorbed into the measurement solution (distilled water). By continuous recording of the conductivity of this solution, oxidation curves were obtained; their inflection point was defined as the induction time (It), which was expressed in hours. Measurements were performed in duplicate.

### 2.10. Statistical Analysis

The analytical determinations were the average of various measurements from independent samples, with statistical differences being estimated by the ANOVA test. The means were compared by the DGC test and the relationship among measured parameters was assessed by Pearson test at a significance level of 0.05 (*p* ≤ 0.05) using Infostat Statistical Software [33].

## 3. Results and Discussion

### 3.1. Porous Starch and Chia Oil Characterization

**Figure 1:**
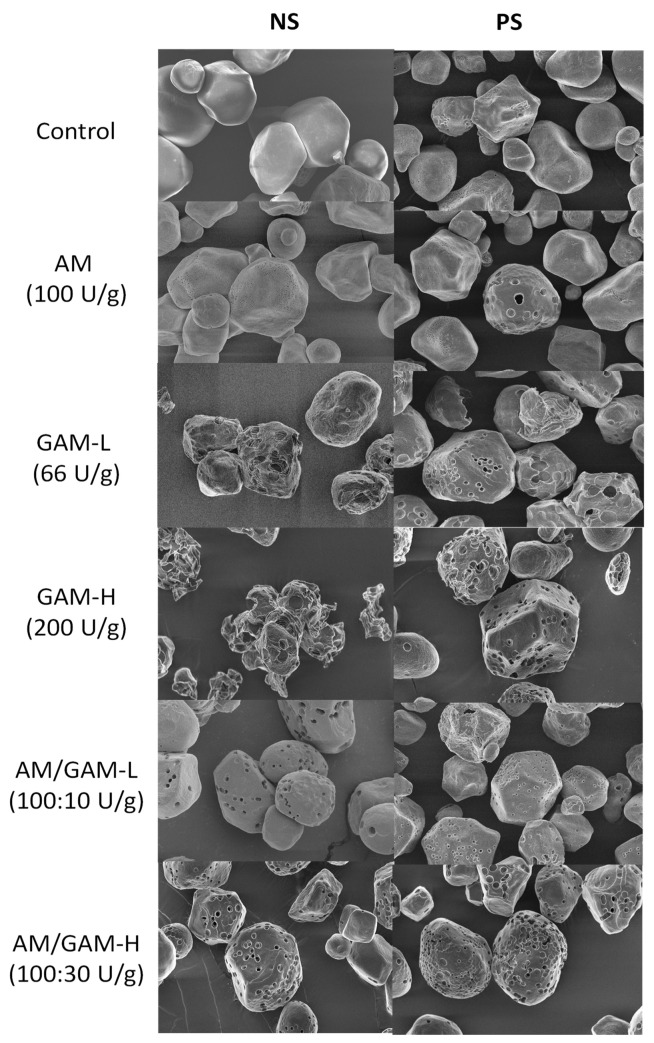
Scanning electron microscopy (SEM) micrographs of native starch (NS) and pretreated starch (PS) with their respective enzymatic treatment: α-amylase (AM), glucoamylase (GAM) and a mixture of both enzymes with two concentrations of GAM (AM/GAM-L and AM/GAM-H). Magnification scale: 2000×.

Figure 1 shows the starch morphology after enzymatic treatments. NS exhibited an irregular shape, mostly polygonal with a very smooth surface [34]. PS kept its granular shape, with less smoothness and some porous structures on the surface due to swelling and heating during pretreatment [35]. The AM treatment produced a very low number of small pores on the granule surface. Similar morphologies have been reported in the literature for other porous starches [34,36,37]. GAM-L and GAM-H treatments formed large and deep pores; yet, these pores were so large that most granules lost their integrity, being potentially unable to introduce and protect bioactive molecules. AM/GAM treatments resulted in a controlled pore production and a more homogeneous pore size and distribution compared with those of GAM treatments. AM/GAM-H conditions showed a higher degree of starch hydrolysis, evidenced by a greater pore number and size in comparison with AM-treated samples. In agreement with our results, Sun et al. [38] reported that AM and GAM act synergistically in starch hydrolysis. Whereas AM acts over the surface of the starch granules, randomly splitting the substrate molecules and exposing non-reducing ends, GAM uses these non-reducing ends to create holes from the surface to the center of the granule, allowing AM to access the inside and generate new sites susceptible to the enzymes.

The effect of the enzymatic treatments on starch size was evaluated; the results are summarized in Table 1. NS values were similar to those of other native starches already reported in the literature [18,20]. PS treatment increased the particle size parameter. This could be due to water penetration into the starch granules during pretreatment, the granules swell and increase in size. Starches treated with GAM (low and high concentration) showed an important decrease in D_v50_, D_4,3_ and D_3,2_, which agrees with the degradation and loss of granule integrity observed through the SEM images.

Table 2 summarizes specific surface area determinations. After enzyme treatment, a significant increase was observed in the specific surface area. The most significant increase in surface area was obtained when GAM was evaluated. This was probably due to the large exposed surface resulting from the granule rupture observed through SEM. A large surface area with granular structure conservation was accomplished with AM/GAM-L and AM/GAM-H treatments. On the other hand, an increase in PS surface area was identified compared with NS. This can be attributed to the swelling experienced by the granules due to pretreatment, which would generate cracks in the granule structure, increasing its specific surface area. After enzymatic treatments, no major differences in surface area were detected between the PS and NS samples.

Table 3 shows the adsorptive capacity of NS and PS samples. PS showed a higher water and sunflower oil adsorption capacity than NS (*p* < 0.0001), probably due to the generation of pores and cracks during pre-treatment that were observed in the SEM images. In agreement with [39,40], the enzymatic treatments increased porous starch matrix adsorption capacities.

Under assay conditions, the highest values were achieved by GAM-L- and GAM-H-treated starches, increasing 60% and 24% for water adsorption capacity and 48% and 30% for sunflower oil adsorption capacity for NS and PS samples, respectively. According to SEM, these samples lost their granular integrity during treatment, which causes a major surface exposure to oil and water, increasing absorption values but making the theme unavailable to protect a bio-active molecule from oxidation or degradation. Starches treated with AM/GAM-H showed higher oil adsorption capacities (19% and 20% for NS and PS samples, respectively), and in particular NS_ AM/GAM-H showed an increase in water adsorptive capacity (35%) in comparison with the untreated starches. These results, along with preserved granular structure observed through SEM, suggest that these materials could be an interesting choice as carriers for bioactive compounds.

Starch thermal properties are summarized in Table 4. It has been established that starch gelatinization involves starch granule hydration and swelling, the irreversible loss of birefringence/crystallinity/granular structure and the melting of crystallite and polymer leaching. It is well known that DSC parameters are correlated with structural changes in the starch crystalline and amorphous regions. According to Evans and Haisman [41], the gelatinization process starts with melting the least stable crystallites (corresponding to T_0_ in DSC endotherms) with subsequent progressive melting into more stable crystallite domains. ΔH reflects the amount of crystalline structure in the granule affected by the thermal treatment. Therefore, lower ΔH values indicate that the starch required less energy to disrupt the polymer and promote gelatinization [39], and higher ΔH values show the opposite.

Enzymatically treated NS, especially with GAM-L and GAM-H, showed higher T_0_ and T_c_ than the untreated sample. These results mean that the proportions of amorphous regions of granules could be reduced as a result of the enzymatic treatment, leading to higher temperatures to disrupt the remaining crystalline regions, which are thermally stable [16,36,42]. ΔH values were similar in the samples, only the NS-AM and NS-AM/GAM-L samples showed small differences with the control (NS). On the other hand, PS had a higher ΔH value than NS (*p* < 0.0001), indicating that pretreatment conditions allowed a rearrangement of the internal structure of starch [43,44]. Enzymatic hydrolysis increased the T_0_ and T_c_ of PS samples; yet, enthalpy was uninfluenced in NS samples.

According to the results, it can be concluded that treatments with AM generated no visible pores in the evaluated starch or significant changes in its water or oil absorption capacity. On the other hand, GAM-treated starch showed a great number of pores and a significant increase in water or oil absorption capacities; however, these samples exhibited a loss of granular structure. However, AM/GAM-H-treated samples yielded the best results, showing homogeneous pore size and distribution, granular structure conservation and significant increment in water or oil absorption capacities. Enzymatically treated samples showed higher gelatinization temperatures, suggesting that enzymes hydrolyze mainly the amorphous regions of starch granules. According to these results, NS and PS treated with AM/GAM-H were selected for further characterization and incorporation of chia oil.

**Figure 2:**
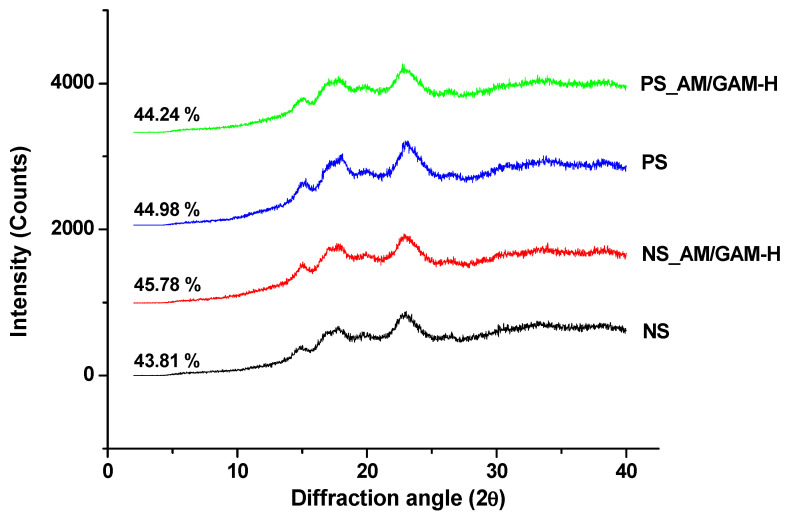
X-ray diffraction (XRD) pattern of starch samples: native starch (NS), pretreated starch (PS) and starch hydrolyzed with the mixture of α-amylase and glucoamylase with the highest dose (NS_AM/GAM-H and PS_AM/GAM-H). Relative crystallinity percentage is shown on the left side of each curve.

The influence of enzymatic treatment was evaluated for crystallinity and the level of damaged starch in the selected samples (NS, PS, NS_AM/GAM-H and PS_AM/GAM-H). Diffraction patterns of the control and AM/GAM-H samples are shown in Figure 2. Samples showed very similar X-ray diffraction patterns, with an A-type pattern characterized by two well-defined peaks at 2 θ values at around 15 and 23° and an unresolved pair of peaks at about 17 and 18° [45]. Relative crystallinity ranged between 43.8 and 45.8% and showed no significant difference among samples (*p* > 0.05) (Figure 2). These results were in agreement with those of the thermal analysis, suggesting that the crystalline structure was not altered by the enzymatic treatment.

Damaged starch content was similar among samples (1.5 ± 0.2%, *p* > 0.05). Similar results were found by Dura et al. [36]. These authors suggested that enzymatic hydrolysis modified the structure of corn starch considerably enough to allow improvement in water and oil adsorption capacity but not rapid enzyme absorption and penetration into the starch structure, as required for the damage starch test. Since the amylase used in the damaged starch determination hydrolyzes mainly amorphous starch, another possible interpretation could include that almost no amorphous starch was rapidly available under the test conditions as a consequence of the enzymatic long exposure time during porous starch preparation with AM and GAM.

Table 5 presents the results obtained from chia oil analysis. Acidity, peroxide index, conjugated dienes and trienes values were similar to some reported in the literature [23,46]. Moreover, the PI value was below the upper limit established by Codex [47] for vegetable oils, demonstrating that the oil extraction process used did not affect the chemical quality of chia oil. Regarding the abundance of polyunsaturated fatty acids, chia oil was composed mainly of linolenic, linoleic and oleic acids, 61.80 g/100 g oil, 20.10 g/100 g oil and 7.18 g/100 g oil, respectively. As expected, a low oxidative stability was observed (2.57 h), mainly due to the fatty acid composition of oil [48].

### 3.2. Oil Incorporation and Product Characterization

**Figure 3:**
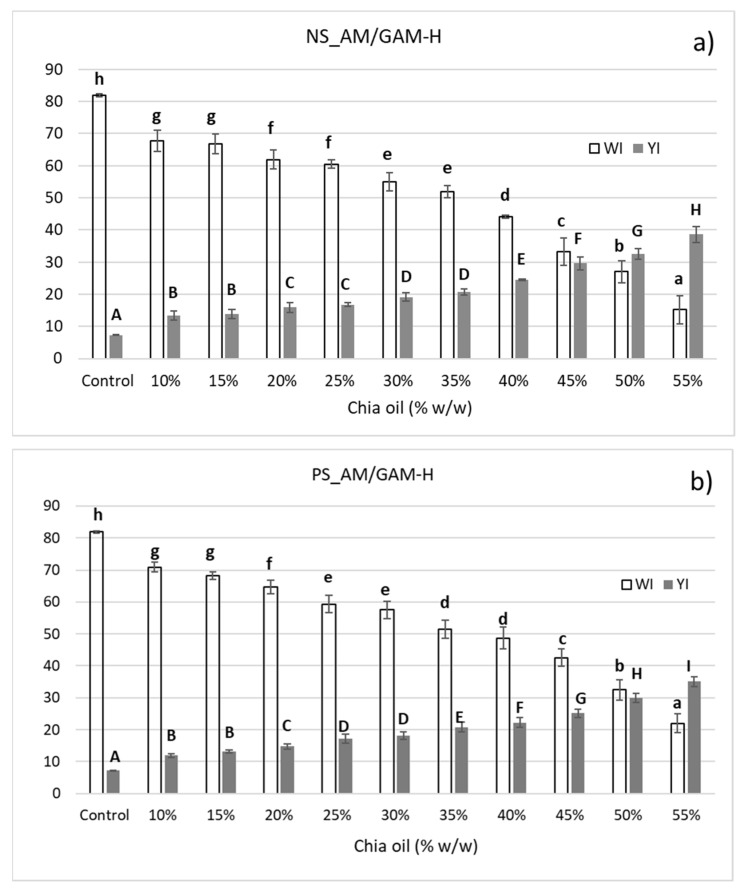
Colorimetric chia-oil-impregnation evaluation of NS (**a**) and PS (**b**) hydrolyzed with the mixture of α-amylase and glucoamylase with the highest dose (AM/GAM-H). WI: whiteness index; YI: yellowness index. Data shown as mean ± SD, *n* = 3. Uppercase and lowercase letters represent the statistical analysis of each sample separately. Means in the same column color followed by the same letter are not significantly different (*p* < 0.05).

Chia oil absorption and protection capacity of the selected porous starch samples (NS_AM/GAM-H and PS_AM/GAM-H) were determined. Chia oil has an intense yellow color, given by pigments such as carotenoids [5]. According to a previous study [7], a decrease in L* and a* parameters and an increase in the b* parameter, i.e., greener and yellower samples, show the incorporation of chia oil. Consequently, an indirect measurement of oil absorption was performed by calculating the color parameters of starch samples (WI: whiteness index and YI: yellowness index) before and after oil impregnation with different proportions of chia oil. In addition, color characterization of the oil-impregnated porous starch is central, considering their potential food application. Color parameters are shown in Figure 3a,b. A decrease and increase of WI and YI, respectively, was observed with the increase in chia oil, suggesting that part of the oil remains on the surface of the starch after the impregnation process and the amount of surface oil is related to the amount of oil in contact with the porous matrix. Above 55%, two well-defined phases were observed for both starches (free-oil phase and starch-oil phase), even after vacuum treatment, indicating that chia oil was no longer able to be absorbed into the porous starches and colorimetric measurements could no longer be performed. The same experiment was carried out for NS as control, with much higher YI (18–38%) and lower WI (15–48%), evidencing the formation of an heterogeneous system with oil percentages superior to 45%, reinforcing the effect of the enzymatic treatment in the starch adsorption capacity (data not shown).

**Figure 4:**
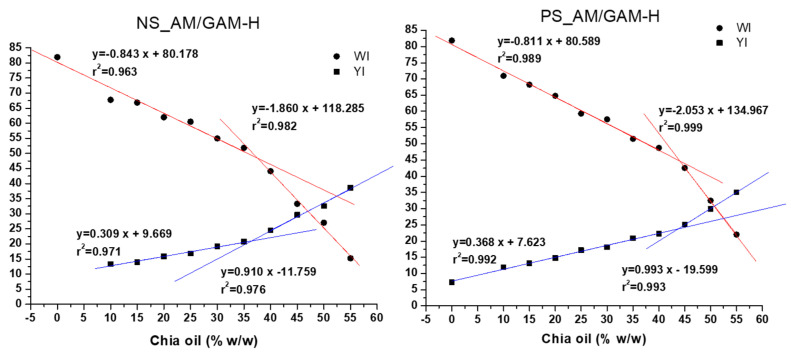
Trend analysis of colorimeter results of native starch (NS) and pretreated starch (PS) hydrolyzed with the mixture of α-amylase and glucoamylase with the highest dose (AM/GAM-H). WI: whiteness index; YI: yellowness index.

Figure 4 shows the WI and YI values (dots and squares) and the fitted linear regression lines of WI and YI versus the percentage of oil used for impregnation (red and blue lines). No significant differences were observed between adsorption capacities of the evaluated porous starches at lower oil percentages (10–25%, *p* < 0.05). For PS_AM/GAM-H, WI values were higher than those for NS_AM/GAM-H, and consequently, YI values were lower at higher impregnation percentages. Moreover, above 35% for NS_AM/GAM-H and 40% for PS_AM/GAM-H, there is an inflection point in the curves and the slopes increase, indicating a possible saturation of pores and the accumulation of oil onto the starch surface. The results suggest that PS_AM/GAM-H was able to adsorb a slightly higher amount of chia oil than NS_AM/GAM-H, due the lower YI, higher WI and the inflection point values of those indexes and that part of the oil remains in the surface after complete saturation of the pores.

**Figure 5:**
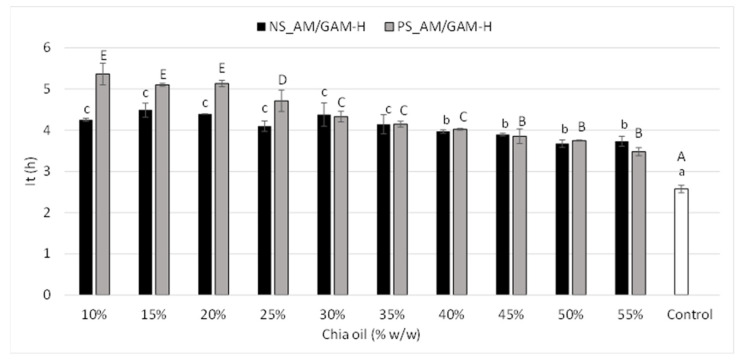
Induction time of impregnated chia oil in native starch (NS) and pretreated starch (PS) hydrolyzed with the mixture of α-amylase and glucoamylase with the highest dose (AM/GAM-H). Data shown as mean ± SD, *n* = 3. Uppercase and lowercase letters represent the statistical analysis of each sample separately. Means in the same column color followed by the same letter are not significantly different (*p* < 0.05).

The impregnated porous starches (NS-AM/GAM-H and PS-AM/GAM-H) and chia oil (control) were exposed to accelerated oxidative conditions using the Rancimat method. This methodology is a useful analytical test not only to measure the oxidative stability of oils but also to select treatments. Results are summarized in Figure 5. Both NS-AM/GAM-H and PS-AM/GAM-H porous starches improved the oxidative stability of chia oil compared with the control, which means that the impregnation into porous starch stabilized the oil and delayed its degradation. Similar results were reported by Gonzalez et al. [7] and Bordón et al. [8] when chia oil was microencapsulated. These authors showed an increase in the induction time of between two and three times for chia oil microencapsulated with isolated soy protein and maltodextrin or gum arabic as wall materials, respectively. It is important to highlight that the PS_AM/GAM-H sample showed significantly higher induction times (near two times the free oil value), especially at lower impregnation percentages (10–25%), evidencing a greater protective effect of the porous starch structure against chia oil oxidation due the impregnation process, in agreement with WI and YI values. The decrease in induction times with the increase of oil in the impregnation process could be linked to the saturation of the starch pores; hence, chia oil remains in the granule surface, increasing its exposure to the degradation process. Finally, when the content of chia oil used in the impregnation process reached 35–40%, no significant differences between the induction times of both porous starches were observed; this is in agreement with the values of the inflection points of color parameters. In addition, a negative correlation for YI (−0.84, *p* < 0.05) and a positive correlation for WI (0.85, *p* < 0.05) with the induction time were determined, indicating that a decrease in YI and an increase in WI are related to an increase in the oxidative stability due to a protective effect of the porous matrix for chia oil.

## 4. Conclusions

Different enzymes and their combinations were evaluated on their ability to produce porous corn starch. Porous starch samples were characterized by SEM, particle size, specific surface area, adsorption capacity and thermal properties. Enzymatic treatments decreased particle size in all the evaluated starches. Enzymatic treatments with AM did not generate visible pores or significant adsorption properties. On the other hand, GAM produced a great number of pores and an important increase in surface area and adsorption properties; however, their granular structure was lost. AM/GAM-H was the condition that yielded the best results, showing a higher number of pores, granular structure conservation and high water and oil adsorption capacities and specific surface area. Regarding thermal properties, all the enzymatically treated samples showed higher gelatinization temperatures, indicating that the enzymes were able to hydrolyze the amorphous part of the starch granules.

Porous starches obtained from NS and PS treated with AM/GAM-H were selected to incorporate and protect chia oil. Color measurements before and after impregnation showed that PS_AM/GAM-H adsorbed more chia oil than NS_AM/GAM-H, reaching a higher saturation point of 40%. Higher induction times were observed for the PS_AM/GAM-H sample, demonstrating greater protection of the porous starch matrix against chia oil oxidation.

No reports have been found on the application of vacuums to facilitate the diffusion of bioactive components in microparticles or in porous starch; thus, this is the first work that describes a procedure to incorporate oil in microporous structures. The results obtained in this work demonstrate the potential application of porous starch obtained by enzymatic hydrolysis for the incorporation and protection of oils susceptible to oxidation. It has also been demonstrated that vacuum impregnation is a potential technique to improve the incorporation of oil in porous structures based on starch.

Further research is needed to improve knowledge of the vacuum impregnation process in porous microstructures. New studies should focus on analyzing what type of mechanisms are involved in the vacuum impregnation process in microstructures, which would allow increasing the amount of oil to be incorporated into these bio-adsorbents and their level of protection.

## Figures and Tables

**Table 1:** Particle size parameters of starch samples.

Sample	D_v10_	D_v50_	D_v90_	[D_4,3_]	[D_3,2_]
NS	8.83 ± 0.08 ^c^	15.77 ± 0.05 ^b^	23.02 ± 0.01 ^b^	15.84 ± 0.04 ^a^	10.62 ± 0.02 ^d^
NS_AM	9.32 ± 0.05 ^d^	16.24 ± 0.05 ^b^	23.81 ± 0.08 ^b^	16.37 ± 0.05 ^a^	11.00 ± 0.02 ^e^
NS_GAM-L	6.97 ± 0.23 ^b^	13.37 ± 0.33 ^a^	21.54 ± 0.22 ^a^	22.28 ± 0.20 ^b^	9.49 ± 0.16 ^b^
NS_GAM-H	6.35 ± 0.03 ^a^	12.81 ± 0.04 ^a^	21.83 ± 0.04 ^a^	22.09 ± 0.81 ^b^	9.23 ± 0.02 ^a^
NS_AM/GAM-L	9.52 ± 0.01 ^d^	15.70 ± 0.42 ^b^	21.96 ± 0.88 ^a^	15.83 ± 0.19 ^a^	10.60 ± 0.07 ^d^
NS_AM/GAM-H	9.19 ± 0.04 ^d^	15.49 ± 0.21 ^b^	21.97 ± 0.40 ^a^	15.43 ± 0.25 ^a^	10.17 ± 0.05 ^c^
PS	10.28 ± 0.13 ^e^	17.32 ± 0.24 ^d^	25.07 ± 0.34 ^e^	17.40 ± 0.25 ^d^	11.49 ± 0.10 ^d^
PS_AM	9.09 ± 0.04 ^c^	15.58 ± 0.05 ^b^	22.31 ± 0.06 ^b^	15.58 ± 0.05 ^b^	10.77 ± 0.02 ^c^
PS_GAM-L	8.69 ± 0.04 ^b^	15.37 ± 0.03 ^b^	23.02 ± 0.06 ^c^	20.46 ± 0.54 ^e^	10.26 ± 0.02 ^b^
PS_GAM-H	6.89 ± 0.04 ^a^	13.18 ± 0.10 ^a^	19.87 ± 0.13 ^a^	13.33 ± 0.09 ^a^	9.48 ± 0.06 ^a^
PS_AM/GAM-L	9.43 ± 0.14 ^d^	16.32 ± 0.19 ^c^	24.13 ± 0.37 ^d^	16.52 ± 0.23 ^c^	10.68 ± 0.09 ^c^
PS_AM/GAM-H	8.72 ± 0.18 ^b^	15.18 ± 0.18 ^b^	22.20 ± 0.16 ^b^	15.29 ± 0.16 ^b^	10.21 ± 0.08 ^b^

Data shown as mean ± SD, *n* = 2. Means in the same column followed by the same letter are not significantly different (*p* < 0.05).

**Table 2:** Specific surface area determination.

Sample	S_BET_ (m^2^/g)
NS	0.2867 ± 0.0093 ^a^
NS_AM	0.5046 ± 0.0038 ^b^
NS_GAM-L	1.0047 ± 0.0142 ^e^
NS_GAM-H	1.0346 ± 0.0180 ^e^
NS_AM/GAM-L	0.7267 ± 0.0102 ^c^
NS_AM/GAM-H	0.8919 ± 0.0097 ^d^
PS	0.4590 ± 0.0055 ^a^
PS_AM	0.5972 ± 0.0064 ^b^
PS_GAM-L	1.0130 ± 0.0096 ^e^
PS_GAM-H	1.1288 ± 0.0066 ^f^
PS_AM/GAM-L	0.7088 ± 0.0121 ^c^
PS_AM/GAM-H	0.8405 ± 0.0052 ^d^

Data shown as mean ± SD, *n* = 2. Means in the same column followed by the same letter are not significantly different (*p* < 0.05).

**Table 3:** Adsorptive capacity of NS and PS starch.

Sample	Adsorptive Capacity (g/g Sample)	
	Water	Sunflower Oil
NS	1.65 ± 0.07 ^aA^	2.12 ± 0.14 ^aA^
NS_AM	1.74 ± 0.04 ^aA^	2.20 ± 0.14 ^bA^
NS_GAM-L	2.09 ± 0.14 ^bB^	2.65 ± 0.18 ^cB^
NS_GAM-H	2.65 ± 0.25 ^eC^	3.15 ± 0.03 ^eC^
NS_AM/GAM-L	2.01 ± 0.21 ^cB^	2.46 ± 0.04 ^cB^
NS_AM/GAM-H	2.22 ± 0.10 ^cB^	2.52 ± 0.06 ^cB^
PS	1.96 ± 0.13 ^bA^	2.15 ± 0.07 ^bA^
PS_AM	1.88 ± 0.10 ^bA^	2.19 ± 0.05 ^bA^
PS_GAM-L	2.25 ± 0.21 ^dB^	2.80 ± 0.13 ^dC^
PS_GAM-H	2.43 ± 0.20 ^cB^	2.81 ± 0.11 ^dC^
PS_AM/GAM-L	2.02 ± 0.24 ^bA^	2.63 ± 0.17 ^cB^
PS_AM/GAM-H	2.10 ± 0.07 ^cA^	2.58 ± 0.03 ^cB^

Data shown as mean ± SD, *n* = 3. Lowercase letters represent the statistical analysis of the total samples; capital letters represent the statistical analysis of each starch sample separately. Means in the same column followed by the same letter are not significantly different (*p* < 0.05).

**Table 4:** Thermal properties determined by DSC of the produced porous starch.

Sample	T_0_ (°C)	T_P_ (°C)	T_c_ (°C)	ΔH (J/g)
NS	66.86 ± 0.17 ^a^	71.70 ± 0.08 ^a^	77.44 ± 0.13 ^a^	9.74 ± 0.01 ^b^
NS_AM	68.84 ± 0.66 ^b^	72.68 ± 0.71 ^b^	78.37 ± 1.20 ^a^	10.85 ± 0.53 ^a^
NS_GAM-L	72.25 ± 0.13 ^c^	76.04 ± 0.18 ^c^	81.60 ± 0.44 ^b^	9.86 ± 0.15 ^b^
NS_GAM-H	74.02 ± 0.10 ^d^	78.12 ± 0.1 ^d^	83.45 ± 0.22 ^c^	9.49 ± 0.11 ^b^
NS_AM/GAM-L	69.22 ± 0.04 ^b^	73.41 ± 0.34 ^b^	79.02 ± 0.57 ^a^	10.72 ± 0.40 ^a^
NS_AM/GAM-H	71.64 ± 0.16 ^c^	75.75 ± 0.28 ^c^	81.29 ± 0.20 ^b^	9.59 ± 0.18 ^b^
PS	66.00 ± 0.32 ^a^	70.59 ± 0.37 ^a^	75.97 ± 0.32 ^a^	11.37 ± 0.01 ^a^
PS_AM	71.41 ± 0.35 ^c^	75.50 ± 0.47 ^c^	81.58 ± 0.76 ^b^	10.91 ± 0.05 ^a^
PS_GAM-L	70.02 ± 0.74 ^b^	74.16 ± 0.85 ^b^	79.84 ± 1.34 ^b^	10.47 ± 0.04 ^a^
PS_GAM-H	71.67 ± 0.22 ^c^	75.89 ± 0.45 ^c^	81.70 ± 0.71 ^b^	10.37 ± 0.05 ^a^
PS_AM/GAM-L	70.21 ± 0.54 ^b^	74.48 ± 0.37 ^b^	80.76 ± 0.21 ^b^	10.81 ± 0.31 ^a^
PS_AM/GAM-H	72.44 ± 0.17 ^c^	76.39 ± 0.21 ^c^	81.98 ± 0.25 ^b^	10.22 ± 0.70 ^a^

T_0_ = onset temperature, T_P_ = peak temperature, T_c_ = endset temperature, ΔH = enthalpy change. Data shown as mean ± SD, *n* = 2. Means in the same column followed by the same letter are not significantly different (*p* < 0.05).

**Table 5:** Chia oil characterization.

Parameter	Value
Acidity (g oleic acid/g oil)	0.33 ± 0.01
Peroxide index (meq O_2_/kg oil)	ND
K232 (conjugated dienes)	1.670 ± 0.100
K270 (conjugated trienes)	0.430 ± 0.050
Induction time (IT)	2.57 ± 0.11
**Fatty Acid (g/100 g oil)**	
Palmitic acid	7.46 ± 0.15
Stearic acid	2.98 ± 0.09
Oleic acid	7.18 ± 0.15
Linoleic acid	20.10 ± 0.16
Linolenic acid	61.80 ± 0.46

Data shown as mean ± SD, *n* = 2.

## Data Availability

The data presented in this study are available on request from the corresponding author.
